# Vacuum levitation and motion control on chip

**DOI:** 10.1038/s41565-024-01677-3

**Published:** 2024-06-06

**Authors:** Bruno Melo, Marc T. Cuairan, Grégoire F. M. Tomassi, Nadine Meyer, Romain Quidant

**Affiliations:** 1 Nanophotonic Systems Laboratory, Department of Mechanical and Process Engineering, ETH Zurich, Zurich, Switzerland; 2 Quantum Center, ETH Zurich, Zurich, Switzerland

**Keywords:** Nanoscience and technology, Optics and photonics, Applied physics

## Abstract

By isolating from the environment and precisely controlling mesoscopic objects, levitation in vacuum has evolved into a versatile technique that has already benefited diverse scientific directions, from force sensing and thermodynamics to materials science and chemistry. It also holds great promise for advancing the study of quantum mechanics in the unexplored macroscopic regime. However, most current levitation platforms are complex and bulky. Recent efforts in miniaturization of vacuum levitation set-ups have comprised electrostatic and optical traps, but robustness is still a concern for integration into confined settings, such as cryostats or portable devices. Here we show levitation and motion control in high vacuum of a silica nanoparticle at the surface of a hybrid optical–electrostatic chip. By combining fibre-based optical trapping and sensitive position detection with cold damping through planar electrodes, we cool the particle motion to a few hundred phonons. We envisage that our fully integrated platform is the starting point for on-chip devices combining integrated photonics and nanophotonics with precisely engineered electric potentials, enhancing control over the particle motion towards complex state preparation and read-out.

## Abstract (web summary)

By combining fibre-based trapping and position detection with cold damping through planar electrodes, cooling of a silica nanoparticle particle motion to a few hundred phonons on a chip is achieved.

## Main

Since the seminal experiment by Ashkin et al., who reported the levitation in vacuum of a microsphere^1^, much progress has been made in controlling the translational and rotational degrees of freedom of levitated objects^2^. Although most efforts have focused on purely optical approaches, more recent developments have evolved towards hybrid platforms combining techniques adapted from atomic physics. For instance, to overcome the constraints posed by intense optical fields in terms of both photodamage and recoil heating^3^, researchers have introduced hybrid optical/electric potentials which combine spatial confinement with high potential depth^4–6^. Furthermore, linear feedback using electric forces on a charged particle^7,8^ enables more efficient cooling compared with its parametric counterpart^9^. Leveraging the palette of available techniques developed over the last decade has enabled the achievement of important milestones, including precision acceleration^10,11^ force^12–16^ and torque^17^ sensing, and achieving ground-state cooling in both one^18–22^ and two dimensions^23^.

As it gains maturity, vacuum levitation is now entering a phase of miniaturization, following the footsteps of trapped ions^24^ and solid-state^25^ systems. Beyond reducing bulkiness and mitigating instabilities associated with multiple assembled parts, on-chip integration is envisioned to facilitate advancements essential for the next generations of experiments. Nanoscale engineering at the chip surface can provide fine control over the electric^26–28^ and magnetic^29^ fields experienced by the particle. It also offers the possibility to enhance the nanoparticle’s interaction with optical fields by exploiting subwavelength modes supported by near-field nanocavities^30^. Interfacing with integrated photonics and meta-optics also has the potential to facilitate scalability towards arrays of multiple traps^31–33^. Finally, on-chip integration provides a pathway towards implementing on a single platform complex dynamic protocols involving bright and dark potentials^34^. Although miniaturization is well underway, with examples such as levitation in planar ion traps^28,35,36^ and optical trapping at the focus of a meta-lens^37^, fully integrated platforms are missing.

Here we present a hybrid photonic–electric on-chip platform enabling the robust levitation, precise position detection and dynamic control of a nanoparticle in vacuum. Our approach circumvents the need for bulky high-numerical-aperture (NA) lenses by combining commercial optical fibres with microscale additive manufacturing to create a robust, versatile and flexible optical interface. Despite the absence of focusing optics, we achieve high signal-to-noise ratios (SNRs) in optical displacement detection that compete with bulky, high-NA optics. When combined with active feedback cooling with planar electrodes, we efficiently cool down the particle motion in three dimensions.

## Integrated hybrid optical–electric chip

**Fig. 1:**
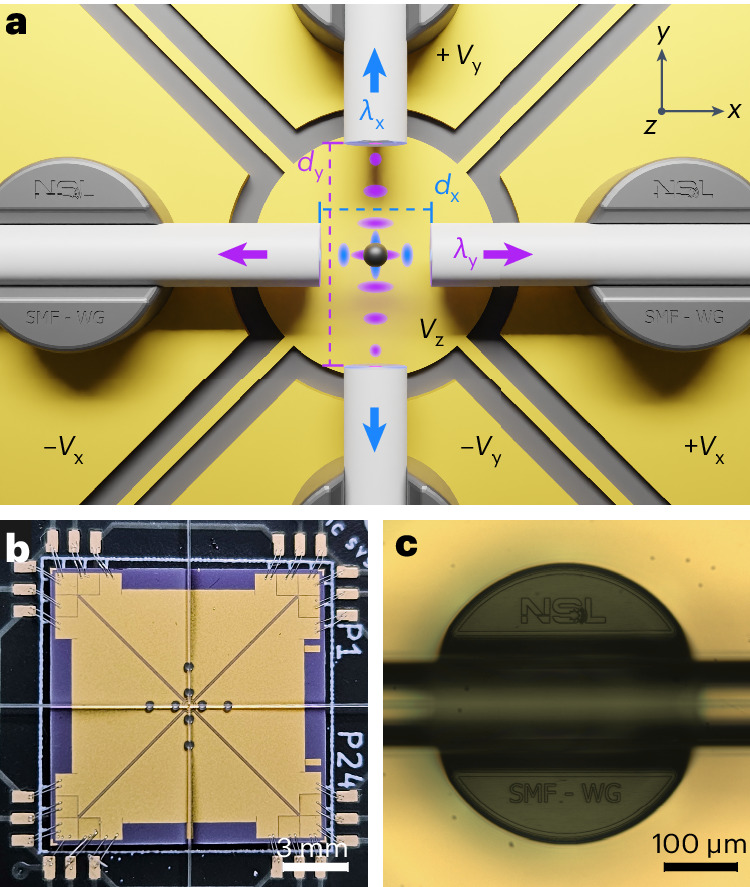
On-chip levitation platform. **a**, The upper optical layer consists of two orthogonal pairs of cleaved single-mode optical fibres. One of the pairs (along *y*) creates a standing wave at *λ*_y_ = 1,550 nm, while the second pair (along *x*) creates a standing wave at *λ*_x_ = 1,064 nm. The distances between the fibres are *d*_x_ = 80 μm and *d*_y_ = 160 μm. A particle (black) is trapped at the intersection of both standing waves. The light scattered by the particle into the fibres, represented by the arrows, is used for displacement detection. The four fibres are positioned above a set of planar electrodes used to apply active feedback cooling to the charged particle via electric forces: right and left electrodes for feedback along *x*, top and bottom for feedback along *y*, and centre electrode for feedback along *z*. **b**, Picture of the levitation chip showing the planar electrodes, four optical fibres, fibre mounts close to the centre and wire bonds from the chip to the PCB at the corners. **c**, Optical fibre positioned into a mechanical mount fabricated via two-photon polymerization and used to align and hold the fibres in place.

Our levitation chip is structured in two layers: an upper photonic layer, where the particle is trapped, allowing precise detection of the nanoparticle’s motion through analysis of the scattered light; and a lower electric layer formed by a set of planar electrodes to cool the particle’s motion. To facilitate direct fibre interfacing, the photonic layer consists of an arrangement of four orthogonal cleaved single-mode optical fibres as illustrated in Fig. 1a. Trapping of the particle occurs in the standing-wave pattern formed by two interfering counterpropagating beams^38,39^. This configuration has the combined advantage of efficiently cancelling the scattering force while creating multiple trapping sites.

Let us first consider one single standing wave (SW) along *y* formed by two equally linearly polarized counterpropagating divergent beams as emerging from two single-mode fibres of NA = 0.1 (refs. ^40,41^), separated by a distance of *d* = 160 μm. The wavelength is *λ*_y_ = 1,550 nm and the total power *P* = 1 W. The equally linearly polarized light field interacts with a nanoparticle of refractive index *n*_r_, radius *R* = 160 nm and polarizability $\alpha =4\uppi {\epsilon }_{0}{R}^{3}({n}_{\mathrm{r}}^{2}-1)/({n}_{\mathrm{r}}^{2}+2)$. At each intensity antinode, the optical force experienced by the particle gives rise to a harmonic potential with theoretical mechanical eigenfrequencies Ω_x,y,z_/(2π) ≈ (3.5, 89, 3.5) kHz and a trap depth *U* = 42*k*_B_*T*_0_, where *k*_B_ is the Boltzmann constant and *T*_0_ = 300 K (room temperature). To achieve three-dimensional active feedback stabilization, it is necessary to ensure well-separated mechanical frequencies along each axis^32^. To this aim, we add a second SW with a wavelength *λ*_x_ = 1,064 nm along the *x* axis, as shown in Fig. 1a. The combination of both SWs results in a high-frequency mechanical mode along each optical axis (*x* and *y*) and a low-frequency mechanical mode (*z*) along the vertical axis.

Beyond ensuring robust trapping above the chip surface and an accurate adjustment of mechanical frequencies, the two pairs of optical fibres serve the additional purpose of monitoring the particle’s position by detecting its scattering. Exploiting the access to both optical axes, the scattered light from each SW is collected by the orthogonal fibre pair (arrows in Fig. 1a) and used to monitor the centre-of-mass (COM) motion. This distinctive collection scheme is inaccessible in single-beam traps and has the advantage of better adjusting to the scattering pattern of the particle^42^.

**Fig. 2:**
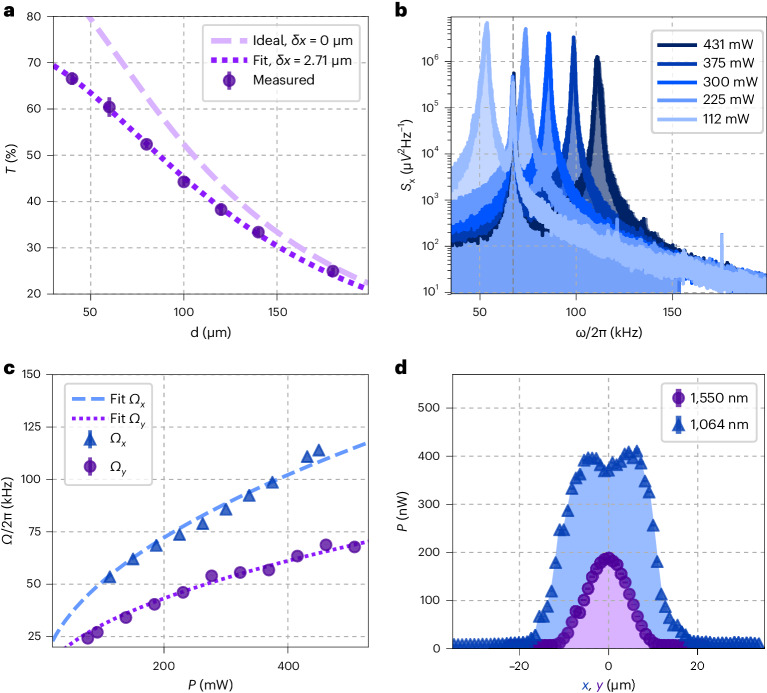
Characterization of the fibre-based trap. **a**, Transmission *T* between two fibres held by the fibre mounts as a function of the distance *d* between the fibres’ end facets. A misalignment of *δx* ≈ 2.71 ± 0.03 μm between the fibres is estimated by fitting the measured transmission (circles) to a theoretical model (dotted line; Supplementary Information, section I). The dashed line shows the theoretical model in the absence of misalignment. Data points and error bars represent the mean value and s.d. of five identically designed fibre pairs. **b**, PSDs of the motion along *x* for different powers *P*_x_ of the 1,064 nm light, while maintaining the 1,550 nm laser power *P*_y_ constant. The trapping powers are estimated from the input power and the coupling and transmission efficiencies throughout the set-up. Each PSD is averaged over 24 PSDs, each based on a 200 ms time trace. **c**, Mechanical eigenfrequencies *Ω*_x,y_ of the motion along *x* (blue triangles) and *y* (purple circles) for different powers of the respective trapping beam. The lines show the fit to the expected behaviour ${{{\varOmega }}}_{\rm{q}}\propto \sqrt{{P}_{\rm{q}}}$. Data points represent the Lorentzian fit to PSDs as shown in **b**. The error bars represent the fitting error. **d**, Power collected by the fibres for different positions (*x*, *y*) of the particle relative to the centre of the collecting fibre. The 1,550 nm (1,064 nm) scattered light is collected by a fibre along *x* (*y*) while the particle is moved along *y* (*x*). The 0 position is defined as the approximate centre of the curves. Data points and error bars represent the mean value and s.d. of 20 identical measurements at a given position.

The fabricated chip measures 0.5 inches × 0.5 inches and is mounted on a custom-made printed circuit board (PCB) for electrical interfacing (Fig. 1b). The electrostatic layer consists of five planar electrodes (Fig. 1a,c), which are used to apply active electrical feedback via electric fields. To achieve reliable levitation, precise control over the position of each cleaved optical fibre is of upmost importance. Consequently, each individual fibre is held in two U-shaped mechanical mounts as shown in Fig. 1c. The latter are microfabricated via two-photon polymerization with a commercial Nanoscribe device (see Chip fabrication and printing of fibre mounts for further details). The relative fibre alignment is assessed by measuring the transmission *T* from fibre to fibre (Thorlabs SMF-28), as shown for 1,550 nm light for different fibre separations *d* in Fig. 2a. By fitting the data (circles) to a theoretical model (Supplementary Information, section I) we extract a relative fibre misalignment of *δx* ≈ 2.71 μm (dotted line). The dashed line shows the ideal case of no misalignment. Once aligned, the cleaved fibres are fixed with epoxy. During the curing process, the transmission *T* varies by a few percent, without significant long-term drifts at constant pressure (Supplementary Information, section I). Nevertheless, *T* consistently increases by around 4% from ambient pressure to vacuum. In general, *T* is stable over time, making this a reliable and robust method to position fibres permanently. Note that our fibre mounting method can be easily employed to create more complex optical lattices due to the arbitrary in-plane positioning of the fibres.

To maximize the trap depth, it is beneficial to position the fibre facets as close as possible to each other. Along *y*, this is limited by the diameter of the fibre cladding (125 μm, Thorlabs 1060XP). Along *x*, *d*_x_ is limited by the diameter of the diverging 1,550 nm beam. To have a safety margin on these constraints and to diminish the interference coming from reflections, we work with *d*_y_ = 160 μm and *d*_x_ = 80 μm. The vertical distance between the nanoparticle and the chip surface is set to 203 μm. No additional fibre treatment or optics are employed for optical trapping.

## Lens-free motional control on chip

Following standard nebulization-based loading, a single silica particle of radius *R* ≈ 160 nm is trapped at the intersection of the two SWs of wavelengths *λ*_x_ = 1,064 nm and *λ*_y_ = 1,550 nm. One distinctive feature of the intersecting SWs lies in the ability to independently tune the mechanical eigenfrequencies Ω_q_ with *q* = *x*, *y*, *z*. As shown in Fig. 2b, by decreasing the power *P*_x_ of the 1,064 nm SW, we observe the expected decrease of Ω_x_, while Ω_y_ remains constant (dashed line). Additionally, by independently varying *P*_x_ and *P*_y_ of each SW and extracting Ω_x_ (purple circles) and Ω_y_ (blue triangles), we verify in Fig. 2c the expected behaviour as ${{{\Omega }}}_{\rm{q}}\propto \sqrt{{P}_{\rm{q}}}$ (dashed and dotted lines). Remarkably, despite the use of low-NA fibres, the achieved values of Ω_q_ are comparable to those produced with high-NA optics.

The particle’s position along *x*, *y* is controlled by changing the relative phase *ϕ*_q_ between the corresponding counterpropagating beams (Supplementary Information, section II). In Fig. 2d, we move the particle along one axis and measure how much light is scattered into the fibre along the perpendicular direction. The blue triangles (purple circles) show the 1,064 nm (1,550 nm) power *P*_x_ (*P*_y_) collected by the fibres along the *y* (*x*) axis. We attribute the shape of the 1,064 nm curve to the multimode character of the Thorlabs SMF-28 fibre at *λ*_x_. To ensure high photon collection efficiency in both directions while maintaining symmetry, we place the particle in the position corresponding to *x*, *y* = 0.

To achieve high feedback efficiency, it is important to consider the angular distribution of the motional information radiation pattern^42,43^. For particles trapped by a single beam, most of the information about the particle’s axial motion is contained in the back-scattered light, enabling one-dimensional ground-state cooling via measurement-based feedback^18,19,22^. In contrast to the single-beam configuration, here the second trapping beam of the SW acts as a strong local oscillator with a fixed phase relation with respect to the back-scattered light. Efficient detection would then require separating the two light fields and using a local oscillator with the appropriate phase.

Instead, the information for the other degrees of freedom (DOF), especially the DOF perpendicular to the polarization axis, is scattered mainly perpendicularly to the beam propagation^42^. In our case, considering a SW along *y* polarized along *z*, the information about the *x* motion is scattered mainly the fibres along *x* (Supplementary Information, section III). This also applies to the *y* motion using the fibre along *y*. Hence, to detect the in-plane motion, we collect the scattered light at *λ*_y_ = 1,550 nm (*λ*_x_ = 1,064 nm) with the fibres along *x* (*y*) and use it in a balanced homodyne scheme to detect the motion along *x* (*y*). To detect the motion along the *z* direction, we use the second fibre along *y*. Here the multimode features of the SMF-28 fibre at *λ*_x_ = 1,064 nm allow the excitation of higher-order modes, increasing the sensitivity to displacements along *z*.

**Fig. 3:**
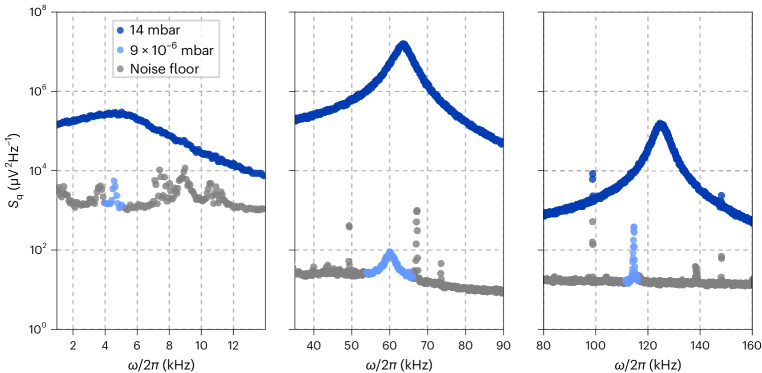
PSDs of the particle’s motion. PSDs of the motion along *z* (left), *y* (middle) and *x* (right), showing resonance frequencies of *Ω*_x,y,z_/(2π) ≈ (120, 65, 5) kHz. At *p* = 14 mbar (dark blue), no feedback cooling is applied. At *p* = 9 × 10^−6^ mbar (light blue), active feedback stabilizes the particle’s motion such that the area of the PSD is decreased. At *ω* ≠ *Ω*_q_ (grey), the detection noise dominates.

The power spectral densities (PSDs) of the motion along *x*, *y*, *z* at a pressure *p* = 14 mbar are plotted in Fig. 3 (dark blue). We reach an optimal SNR_y_ ≈ 10^6^, comparable to forward-scattering detection with high-NA optics in standard experiments^44^.

To cool and stabilize the nanoparticle’s COM motion in vacuum, we apply electrical cold damping^7,8^ along *q* = *x*, *y*, *z*. The nanoparticle’s COM motion is modelled as three decoupled harmonic oscillators described by the equation of motion

1 $$\ddot{q}(t)+{{{\varGamma }}}_{\mathrm{m}}\dot{q}(t)+{{{\varOmega }}}_{\rm{q}}^{2}q(t)=\frac{{F}_{\mathrm{th}}(t)+{F}_{\rm{q}}^{{{\,{\rm{fb}}}}}(t)}{m},$$

where *m* is the oscillator’s mass, *Γ*_m_ is the mechanical damping rate due to the surrounding gas, leading to a stochastic force $${F}_{\mathrm{th}}(t)=$$$$\sigma \eta (t)=\sqrt{2{k}_{\mathrm{B}}Tm{{{\varGamma }}}_{\mathrm{m}}}\eta (t)$$ with *η*(*t*) being white noise with unit standard deviation and zero mean^45^. The feedback force is proportional to the delayed position, that is, $${F}_{\rm{q}}^{{{\,{\rm{fb}}}}}(t)\propto {k}_{\rm{q}}^{\rm,d}\hspace{2.22144pt}q(t-\tau )$$ where $${k}_{\rm{q}}^{\rm,d}$$ is an adjustable gain and *τ* is a tunable delay. For *τ* = π/(2*Ω*_q_) this leads to an effective damping rate $${{{\varGamma }}}_{\rm{q}}^{{{{\rm{eff}}}}}={{{\varGamma }}}_{\mathrm{m}}+{{{\varGamma }}}_{\rm{q}}^{{{{\mathrm{fb}}}}}$$ optimized for cooling^7^.

The externally applied feedback force ${F}_{\rm{q}}^{{{\,{\rm{fb}}}}}(t)=Q{E}_{\rm{q}}(t)$ depends on the charge of the particle *Q* = *n*_q_*e*^−^ with *n*_q_ elementary charges and the homogeneous electric field *E*_q_(*t*) ∝ ±*V*_q_(*t*) generated by planar electrodes as shown in Fig. 1a. For the in-plane DOF *x*(*y*), we apply two voltage signals ±*V*_x_(*t*) (±*V*_y_(*t*)) of equal amplitude but out of phase to the pair of electrodes situated left and right (top and bottom) of the chip. For cooling the *z* direction, we apply *V*_z_(*t*) to a single planar electrode depicted in the centre of Fig. 1a. The symmetric electrode layout and the electrode–particle distance ensure the homogeneity of *E*_q_(*t*) (Supplementary Information, section II).

The results of 3D cold damping on chip under a vacuum are shown in Fig. 3. The individual panels display the Lorentzian PSDs of the particle displacement ${S}_{\rm{q}}^{{{{\rm{IL}}}}}(\omega )$ at pressures *p* = 14 mbar (dark blue) and *p* = 9 × 10^−6^ mbar (light blue). In the latter, we stabilize the particle using active feedback in 3D.

## Cooling and sensing performance

The lowest temperature ${T}_{\rm{q}}^{{{\,{\rm{eff}}}}}$ achievable in cold damping is determined by the detection noise *σ*_q_ and the mechanical damping *Γ*_m_ ∝ *p* (ref. ^46^). We fit the PSD of the in-loop (IL) detector^7^

2 $$\begin{array}{l}{S}_{\rm{q}}^{{{{\rm{IL}}}}}(\omega )=\frac{{\sigma }^{2}/{m}^{2}}{{\left({{{\varOmega }}}_{\rm{q}}^{2}\,-\,{\omega }^{2}\right)}^{2}+{\left({{{\varGamma }}}_{\rm{m}}+{{{\varGamma }}}_{\rm{q}}^{{{{\rm{fb}}}}}\right)}^{2}{\omega }^{2}}\\\qquad\quad+\,\frac{{\left({{{\varOmega }}}_{\rm{q}}^{2}\,-\,{\omega }^{2}\right)}^{2}+{{{\varGamma }}}_{\rm{m}}^{2}{\omega }^{2}}{{\left({{{\varOmega }}}_{\rm{q}}^{2}\,-\,{\omega }^{2}\right)}^{2}+{\left({{{\varGamma }}}_{\rm{m}}+{{{\varGamma }}}_{\rm{q}}^{{{{\rm{fb}}}}}\right)}^{2}{\omega }^{2}}{\sigma }_{\rm{q}}^{2}\end{array}$$

to our data (Supplementary Information, section V). This allows us to determine the oscillator’s effective COM temperature ${T}_{\rm{q}}^{{{\,{\rm{eff}}}}}$ as

3 $${T}_{\rm{q}}^{{{\,{\rm{eff}}}}}=\frac{m{{{\Omega }}}_{\rm{q}}^{2}}{2{k}_{\rm{B}}}\left(\frac{{\sigma }^{2}/{m}^{2}}{{{{\Omega }}}_{q}^{2}\left({{{\Gamma }}}_{m}+{{{\Gamma }}}_{\rm{q}}^{{{{\rm{fb}}}}}\right)}+\frac{{\left({{{\Gamma }}}_{\rm{q}}^{{{{\rm{fb}}}}}{\sigma }_{\rm{q}}\right)}^{2}}{{{{\Gamma }}}_{\rm{m}}+{{{\Gamma }}}_{\rm{q}}^{{{{\rm{fb}}}}}}\right),$$

where ${{{\varGamma }}}_{\rm{q}}^{{{{\rm{fb}}}}}\propto \,{k}_{\rm{q}}^{\rm,d}$. The phonon occupation then is

4 $$\langle {n}_{\rm{q}}\rangle =\frac{{k}_{\mathrm{B}}{T}_{\rm{q}}^{{{\,{\rm{eff}}}}}}{\hslash {{{\varOmega }}}_{\rm{q}}}$$

**Fig. 4:**
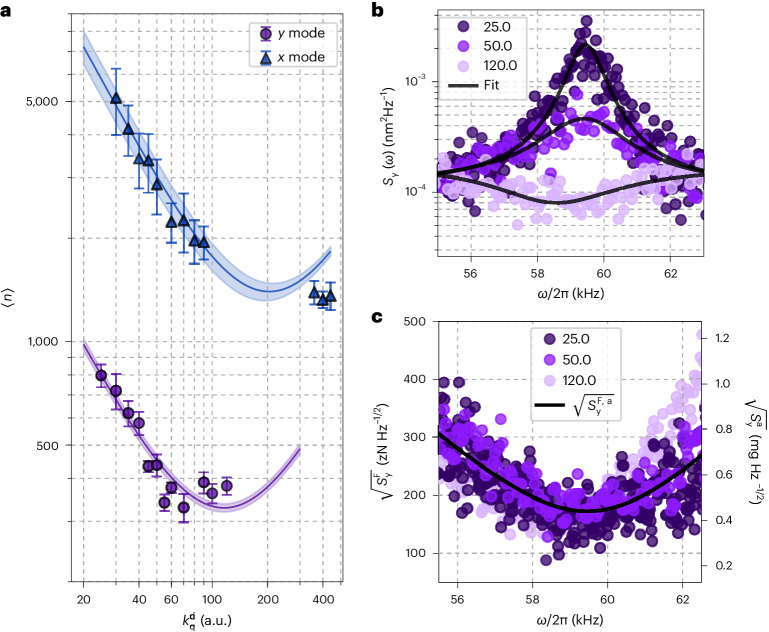
Feedback cooling and sensitivity. **a**, Mean phonon occupation of the motion 〈*n*_q_〉 along *x* (blue triangles) and *y* (purple circles) for increasing feedback gains ${k}_{\rm{q}}^{\rm,d}$, extracted from the in-loop PSDs. The data points and error bars are the mean and s.d. of ten independent temperature measurements. The solid lines are fits to equation (4) with ${T}_{\rm{q}}^{{{\,{\rm{eff}}}}}$ given by equation (3) where ${{{\varGamma }}}_{\rm{q}}^{{{\,{\rm{fb}}}}}$ is the only free parameter. Other parameters are extracted from the in-loop PSDs. The shaded regions correspond to the uncertainty of the extracted parameters (Supplementary Information, section VI). **b**, PSD of the *y* motion for three different values of the feedback gain. The solid lines are fits to equation (2) (see text for details). **c**, PSDs of the force and acceleration sensitivities for the same three feedback gains as in **b**. The solid line is the minimum force and acceleration sensitivity from equations (5) and (6).

Figure 4a displays the estimated phonon occupation 〈*n*_q_〉 for increasing feedback gain ${k}\,_{\rm{q}}^{\rm{d}}$ along the *x* (blue triangle) and *y* (purple circles) directions. The phonon occupation decreases to 〈*n*_y_〉 = 329 ± 30 and 〈*n*_x_〉 = 1,325 ± 72, respectively. The solid lines represent fits to equation (4) with the shaded regions indicating the uncertainty associated with the fitted curve (Supplementary Information, section VI). For large feedback gains, 〈*n*_q_〉 increases due to correlations between the detector noise *σ*_q_ and feedback signal, as depicted in Fig. 4a. In the PSD, it manifests as so-called noise squashing^7,8,47^, such that ${S}_{\rm{q}}^{{{{\rm{IL}}}}}({{{\varOmega }}}_{\rm{q}})$ yields underestimated values below the noise floor. This is depicted in Fig. 4b, where we plot the measured PSD of the *y* motion for different feedback gain values. Note that we use the in-loop detection signal for the temperature estimation. This approach remains valid, given that we account for the noise squashing by adding the second term in equation (3)^7^, making an out-of-loop detector unnecessary for the temperature estimation.

Due to its compact design and excellent cooling performance, the developed platform shows promise for application in force and acceleration sensing. The minimum force and acceleration sensitivity taking into account detection noise (Supplementary Information, section VII) exceeds the thermal limit and is given by

5 $$\sqrt{{S}_{\rm{q}}^{\mathrm{F}}}=\sqrt{2m{k}_{\mathrm{B}}T{{{\varGamma }}}_{\mathrm{m}}+{m}^{2}\left[{\left({{{\varOmega }}}_{\rm{q}}^{2}-{\omega }^{2}\right)}^{2}+{\omega }^{2}{{{\varGamma }}}_{\mathrm{m}}^{2}\right]{\sigma }_{\rm{q}}^{2}}$$

6 $$\sqrt{{S}_{\rm{q}}^{\mathrm{a}}}=\sqrt{2{k}_{\mathrm{B}}T{{{\varGamma }}}_{\mathrm{m}}/m+\left[{\left({{{\varOmega }}}_{\rm{q}}^{2}-{\omega }^{2}\right)}^{2}+{\omega }^{2}{{{\varGamma }}}_{\mathrm{m}}^{2}\right]{\sigma }_{\rm{q}}^{2}}$$

and is independent of the cooling of the COM. Yet, for real-life applications, the detection noise *σ*_q_ limits the bandwidth in which minimal sensitivities are achievable.

In Fig. 4c, we display the force and acceleration spectral densities, $\sqrt{{S}_{\rm{y}}^{\mathrm{F}}}$ and $\sqrt{{S}_{\rm{y}}^{\mathrm{a}}}$, for a range of ${k}_{\rm{y}}^{\rm,d}=[25,50,120]$. The solid line corresponds to equations (5) and (6) using the parameters extracted from the fitted in-loop PSDs. The experimentally achieved minimum sensitivities are $\sqrt{{S}_{\rm{y}}^{\mathrm{F}}}=176\pm 26\,{\mathrm{zN}}\,{\mathrm{Hz}}^{-1/2}$ and $\sqrt{{S}_{\rm{y}}^{\rm{a}}}=440\pm 70\,\upmu {\mathrm{g}}\,{\mathrm{Hz}}^{-1/2}$ which increase by 3 dB over an 8 kHz bandwidth around *Ω*_y_/(2π) = 59 kHz due to detector noise contributions exceeding the thermal noise. As expected, both bandwidth and sensitivities show no dependence of ${k}_{\rm{y}}^{\rm,d}$. Note that the hypothetical additional use of an out-of-loop detector would further deteriorate the sensitivities because we would have noise contributions from both detectors (Supplementary Information, section VII).

## Conclusions

We have demonstrated robust optical on-chip levitation and motion control in vacuum with a fully integrated platform. Despite the use of commercial fibres with low NAs, we have shown that the particle’s displacement detection reaches comparable SNRs as with bulky high-NA optics. Such performance, which already enables the cooling of the COM down to hundreds of phonons, can be enhanced even more by further reducing the fibre distance.

The implemented platform also offers interesting applications towards multiparticle arrays^32^ beyond ten particles, optical binding^31,48^ and the levitation of high-refractive-index meta-atoms^49^. Additionally, as previously mentioned, our fabrication technique also enables more complex lattice forms^50^ by controlled in-plane fibre positioning.

Furthermore, the microfabricated fibre mounts are well suited for integrating a more controlled particle-loading mechanism^51,52^. By replacing one of the single-mode fibres with a hollow core fibre, individual particles could be delivered to the trapping region using an optical conveyor belt. To date this appears to be the only technique that offers deterministic particle loading into optical traps in ultrahigh-vacuum conditions^53^—an important prerequisite for the generation of macroscopic quantum states^34,54^.

In future versions of the platform, the integration of refractive microlenses^55^ or metalenses^56,57^ at the output facet of the fibres promises to enhance both detection sensitivity and achievable mechanical frequencies along the *z* axis. Furthermore, more complex optical elements such as fibre cavities^58,59^ can also be integrated. We envision our platform as the initial stepping stone towards the use of hybrid potentials for quantum experiments based on levitated particles.

## Methods

### Chip fabrication and printing of fibre mounts

**Extended Data Fig. 1:**
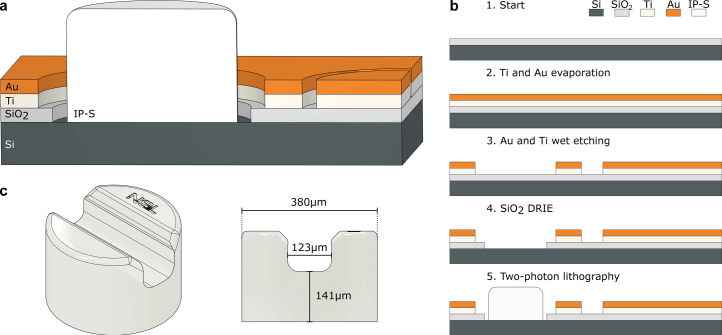
Fabrication of the on-chip platform. **a**) The substrate is made of silicon covered with a layer of silicon oxide to isolate the substrate from the electrodes. The electrodes are made of gold, and a titanium layer is used to increase the adhesion of the electrodes to the silicon oxide. The fibre mounts are fabricated directly onto the silicon. **b**) Summarized fabrication process: 1. We start from a silicon wafer with a layer of silicon oxide. 2. The titanium and the gold are evaporated on the wafer. 3. The gold and the titanium are etched to create the electrodes. 4. The silicon oxide is etched to created the clearance for the fibre mounts. 5. After dicing the wafer, the fibre mounts are printed onto the silicon via two-photon polymerization. **c**) Fibre mounts used to align the fibres in our chip. The width of the square slot accommodating the fibre is chosen to be slightly smaller than the cladding of the single mode fibres.

The layer structure of our chip and the fabrication steps are illustrated in Extended Data Fig. 1. We start with a 530-μm-thick silicon 4 inch wafer with a 500-nm-thick silicon dioxide (SiO_2_) layer. The 500 nm oxide layer serves as an insulator layer to prevent electrical breakdown between the electrodes and the bulk silicon for voltages up to 235 V. Next, we evaporate a 5-nm-thick adhesion layer of titanium and a 100-nm-thick layer of gold.

To create the electrode pattern, the wafer is first spin coated with photoresist (AZ1512, 40 s at 4,000 rpm), baked for 60 s at *T* = 110 °C and exposed to a first mask using ultraviolet light. The resist is then developed and baked at *T* = 135 °C for 3 min. Then the gold is etched (TFA, Transene) for around 30 s, and subsequently the titanium is etched in hot hydrochloric acid (*T* = 90 °C) for around 60 s. The resist is then stripped off using acetone.

The next step is to etch the silicon oxide layer to create the clearance for printing the fibre mounts directly on silicon. We first spin coat the wafer with photoresist (AZ4562, 40 s at 1,500 rpm, resulting in a thickness of around 10 μm) and then bake it for 5 min at *T* = 110 °C. This is followed by a second round of mask exposure using ultraviolet light and development of the resist. The oxide is then etched using reactive-ion etching with fluoroform (CHF_3_) and argon for 33 min at 75 W. The resist is then stripped off using dimethylsulfoxide at 90 °C. Once the electrodes are patterned and the silicon oxide is etched, the wafer is diced into 0.5 inch × 0.5 inch samples.

The fibre mounts, shown in Fig. 1c, are then printed on each sample individually via dip-in two-photon lithography with a femtosecond laser lithography system (Photonic Professional GT, Nanoscribe). To increase the adhesion of the exposed IP-S resist (Nanoscribe) to the silicon subtrate we activate the surface using oxygen plasma (200 W for 30 s) and silanize it by immersing the chip into a mixture of 30 ml ethanol, 150 μl 3-(trimethoxysilyl)propyl methacrylate and 1.5 ml of acetic acid (1:10 acetic acid:water) for 30 min prior to the two-photon lithography. For printing, we use IP-S resist in combination with a 25× objective lens, which provides a printing area 400 μm in diameter. After the resist is exposed, the sample is developed in propylene glycol methyl ether acetate for 20 min and then rinsed with isopropyl alcohol for 3 min. Finally, the sample is glued to a PCB and the electrodes are connected to the board via wirebonding.

## Online content

Any methods, additional references, Nature Portfolio reporting summaries, source data, extended data, supplementary information, acknowledgements, peer review information; details of author contributions and competing interests; and statements of data and code availability are available at 10.1038/s41565-024-01677-3.

## Supplementary information

Supplementary Information Supplementary sections I–VII and Figs. 1–7.

## Data Availability

Source data for Figs. 2, 3 and 4 are available via the ETH Zürich Research Collection at 10.3929/ethz-b-000667724.
