# Supplementary material for: Vacuum levitation and motion control on chip
Source: Nat Nanotechnol. 2024 Jun 6;19(9):1270–6. doi: 10.1038/s41565-024-01677-3 (PMC11405270; doi:10.1038/s41565-024-01677-3)
Supplement: Supplementary file 1 — Supplementary sections I–VII and Figs. 1–7. [file 41565_2024_1677_MOESM1_ESM.pdf]

nature nanotechnology

Supplementary information https://doi.org/10.1038/s41565-024-01677-3

Vacuum levitation and motion control on chip

In the format provided by the authors and unedited

CONTENTS

I. Alignment characterization 1

II. Experimental setup 1

III. Information scattering 2

IV. Cold damping and temperature measurements 4

V. Calibration and data analysis 7
1. Calibration of the particle displacement 7
2. Extracting the center-of-mass effective temperature 7

VI. Sensing with a cold damped harmonic oscillator 7

VII. Surface noise heating 9

References 10

I. ALIGNMENT CHARACTERIZATION

To characterize the alignment provided by the micro fabricated fiber mounts, we measure the light transmission from fiber to fiber as a function of the fiber distance. To measure this transmission, we place fiber (Thorlabs SMF28) in a pair of fiber mounts and measure the output power P_0. A second fiber is placed on another pair of fiber mounts facing the first fiber. The power coupled into the second fiber is measured with a power meter for different separations d between the fiber facets. This is repeated for five sets of fiber mounts to produce the mean values and the error bars in Fig. 2a.

The measured values are compared to a theoretical model, which assumes that the output of our single mode fibers is approximately Gaussian. Under this assumption, the electric field launched by a fiber along y is:

$$\vec{E}_{\pm y}(\vec{r}) = \sqrt{\frac{4P_{0,y}}{\pi c\epsilon_0 w_{0,y}^2}}\frac{w_{0,y}}{w_y(y)}e^{-\frac{(x^2+z^2)}{w_y(y)^2}}\exp\left(\pm ik_y(y-y_{0,\pm}) \mp i\arctan\frac{y-y_{0,\pm}}{y_R} \pm i\frac{k_y(x^2+z^2)}{2}\frac{y-y_{0,\pm}}{(y-y_{0,\pm})^2+y_R^2}\right)\hat{u}_y \quad (1)$$

where $P_{0,y}$ is the power of the beam, c the speed of light, ϵ_0 the electric permittivity of vacuum, $w_{0,y}$ the waist of the beam at the output of the fiber (assumed to be equal to half of the mode field diameter of the fiber), $y_R = \pi w_{0,y}^2/\lambda_y$ the Rayleigh range, $w_y(y) = w_{0,y}\sqrt{1+(y-y_{0,\pm})^2/y_R^2}$ the waist of the beam at the position y, $k_y = 2\pi/\lambda_y$ the wave number, $y_{0,\pm}$ is the position of the fiber facet and $\hat{u}_y$ is the unit vector of the polarization direction. The $+$ and $-$ signs apply if the fiber is pointing in the positive or negative direction, respectively. For the fibers along the x direction, we exchange x and y.

In our model, we consider the first fiber to be positioned at the origin, while the second is displaced axially by a distance d and radially by a distance δx. The transmission is then given by the overlap integral between the field from the first fiber after propagating through the distance d and the fundamental mode of the second fiber:

$$T(d,\delta x) = 0.96 \times \frac{\left|\int\int E_{+y}(x,d,z)E_{+y}^*(x-\delta x,0,z)dxdz\right|^2}{\int\int |E_{+y}(x,d,z)|^2\, dxdz \int\int |E_{+y}(x-\delta x,0,z)|^2\, dxdz} \quad (2)$$

where the 0.96 factor accounts for the reflection at the air-fiber interface encountered by the beam when entering the second fiber. Note that, in practice, the displacement along x could be an offset between the fiber's center in any radial direction.

It is also relevant to look at how this transmission varies once the fibers are glued in place with epoxy. The normalized transmission during the curing process is shown in Fig. S1a. At the time $t = 0$ the epoxy is applied and left to cure. As we can see, the change in transmission over the initial 12 hours is smaller than 4%. After the curing process is complete, the transmission gets more stable, as shown in Fig. S1b, where variations smaller than 2% are observed over a period of 24 hours. Finally, we monitor the transmission as a function of pressure when going from ambient pressure to 5×10^{-6} mbar. The result is displayed in Fig. S1c, where a change of around 3% is observed between 10^{-2} mbar and 10^{-4} mbar.

II. EXPERIMENTAL SETUP

The schematic of the experimental setup is shown in Fig. S2. We trap a silica nanoparticle in the center of two perpendicular standing waves at wavelengths $\lambda_x = 1064$ nm and $\lambda_y = 1550$ nm. In Fig. S2a (top) we show the optical setup used to generate the standing wave trap at $\lambda_x = 1064$ nm (depicted in blue). The laser light is split in two beams using a polarizing beam splitter (PBS), and the power in each beam is controlled by adjusting the polarization

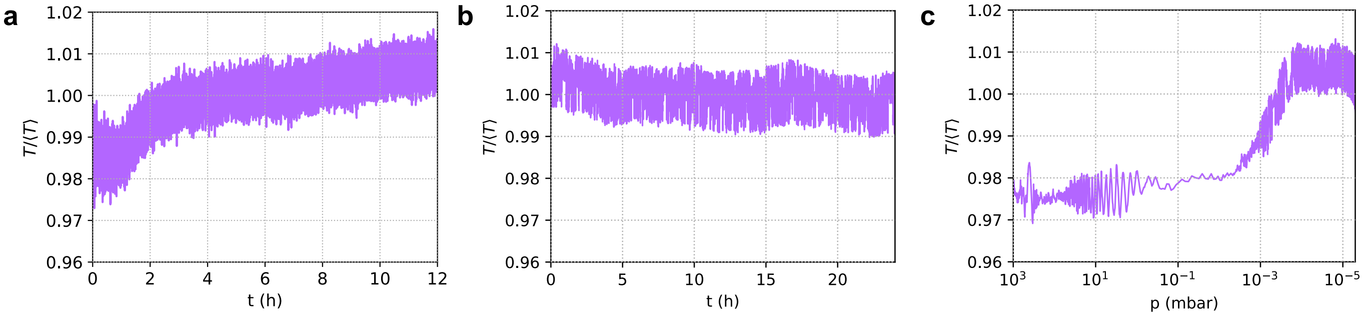

Figure S1. **Characterization of the fiber alignment. a)** Transmission from one fiber to another after they are positioned inside the fiber mounts and the epoxy is applied and left to cure. **b)** Transmission from one fiber to another at ambient pressure after the epoxy is cured. **c)** Transmission from one fiber to another when the pressure is reduced from ambient pressure to 5×10^{-6} mbar. In all plots the transmission is normalized by the average value over the period of measurement.

using a half-waveplate ($\lambda/2$). One of the beams is directly coupled to a fiber (Fiber x1). The second beam is reflected by a mirror with a piezo element attached to it to adjust the beam path and then coupled to the second fiber (Fiber x2). We use polarization optics in front of the fiber couplers to independently adjust the polarization of the two beams and maximize the interference.

By controlling the voltage sent to the aforementioned piezo element we can move the positions of the nodes of the standing wave or lock the relative phase between the two beams. To lock the relative phase we interfere the light transmitted through the chip (from Fiber x1 to Fiber x2) with the light reflected from the fiber's facet (Fiber x2) and lock to a chosen intensity value using a P-controller implemented with a Field Programmable Gate Array (FPGA) (ϕ-Lock, Red Pitaya). The same applies to the generation of the standing wave at $\lambda_y = 1550$ nm (depicted in purple) Fig. S2a (bottom).

The $\lambda_x = 1064$ nm light scattered by the particle is collected using Fiber y1 and Fiber y2 and used for detecting the motion along y and z, respectively. The $\lambda_y = 1550$ nm scattered light is collected using Fiber x2 and used for detecting the motion along x. For the x and y detection, the collected light is sent to their respective balanced homodyne detection. For the z detection the light is sent to a photo detector. In Fig. S2b, we show the schematics of our balanced homodyne detection. The signal (collected scattered light) is reflected by a mirror on a piezo actuator to control the beam path, combined with a local oscillator using a beam splitter (BS) and sent to a balanced detector (Newport 1870-FC). We actively stabilize the homodyne phase by actuating on the voltage sent to the piezo actuator using a P-controller implemented with an FPGA.

The homodyne signal $q(t)$ is sent to an FPGA to create the feedback signal $V_q(t)$ sent to the corresponding planar electrode for stabilizing the center of mass motion using cold damping. To create electric fields along the three directions we use a set of 5 electrodes, as depicted in Fig S2c. Fig S2d-i show the electric field generated by activating different electrodes: S2d-e show the simulated electric field along x generated by the x electrodes, S2f-g the electric field along y generated by the y electrodes and S2h-i the electric field along z generated by the z electrode. ± 1 V is applied to the respective electrodes. In the vicinity of the center, the electric field is approximately constant, with variations smaller than 0.5% over a region of $5\mu\text{m} \times 5\mu\text{m} \times 5\mu\text{m}$ around the origin, justifying the assumption of a homogeneous electric field at the particle's position.

III. INFORMATION SCATTERING

Here we analyze how a dielectric particle trapped at the origin by a standing wave along x with linear polarization along z scatters information about its motion along y. We follow an approach similar to the one in [1].

The total field at the vicinity of the origin is, to first order in x, y, z:

$$\vec{E}_{total}(\vec{r}) = \vec{E}_{+x}(\vec{r}) + \vec{E}_{-x}(\vec{r}) \approx \sqrt{\frac{4P_{0,x}}{\pi c \epsilon_0 w_{0,x}^2}} \left(e^{iAk_x(x-x_{0,+})} + e^{-iAk_x(x-x_{0,-})} \right) \hat{z} \tag{3}$$

where Ak_x is the effective wave number due to the Gouy phase.

The total electric field scattered by a particle at $\vec{r}_p$ at a coordinate $\vec{r} = (r, \theta, \phi)$ far from the particle is:

$$\vec{E}_{sc}(\vec{r}) = \vec{E}_1(r,\theta,\phi) e^{iAk_x(x-x_{0,+}) - ik_x \vec{r}_p \cdot \vec{n}_{\vec{r}}} + \vec{E}_2(r,\theta,\phi) e^{-iAk_x(x-x_{0,-}) - ik_x \vec{r}_p \cdot \vec{n}_{\vec{r}}} \tag{4}$$

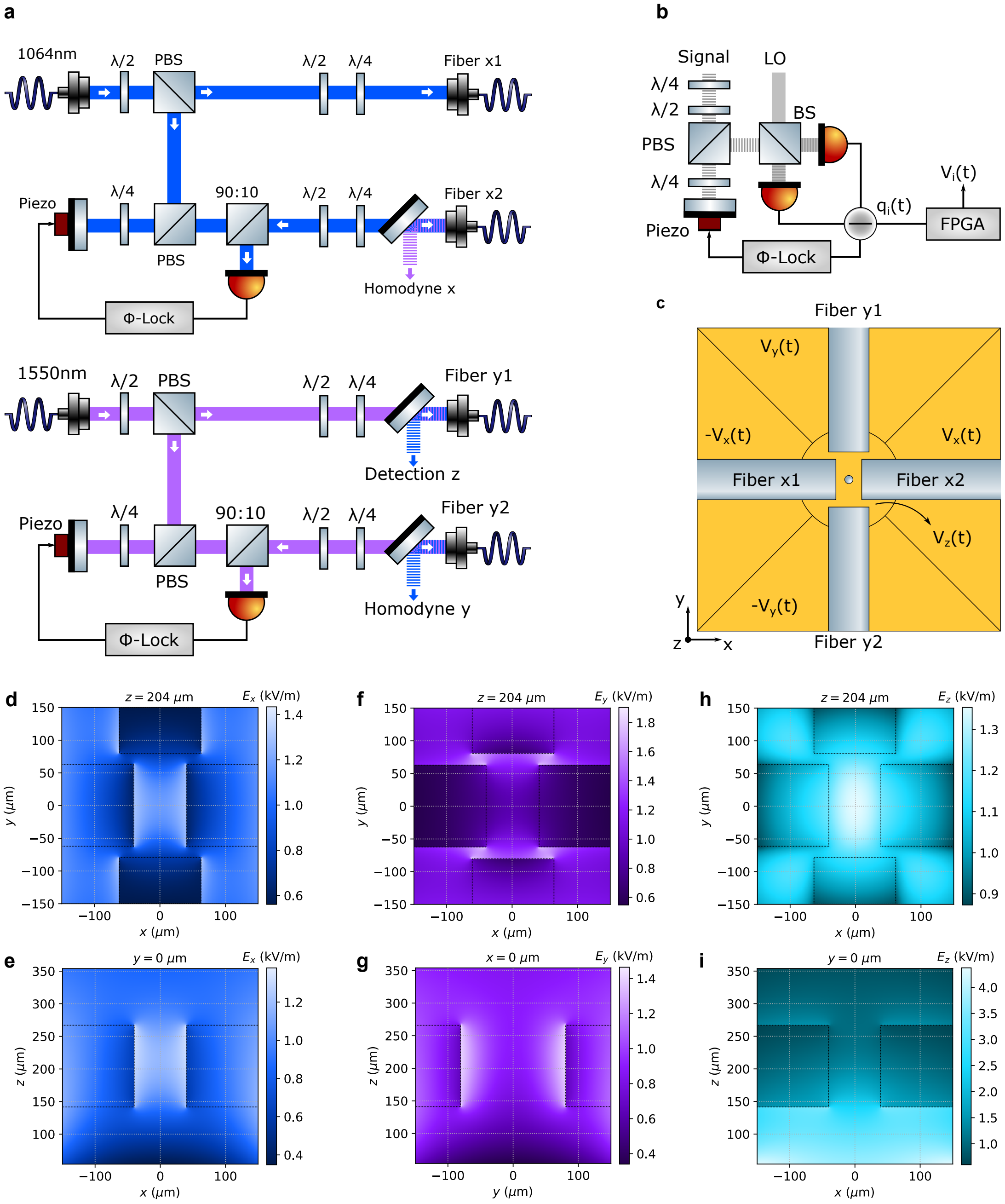

Figure S2. **Experimental setup for trapping, detection and cooling.** **a)** Setup used for preparing the trapping beams. The light from a laser source is split in two beams using a PBS. One of the beams goes directly to the chip, while the other is first reflected by a mirror on a piezo actuator before going to the chip. The polarization of each beam can be independently controlled to maximize the interference at the trapping region. The relative phase between the beams can be adjusted using the piezo actuator and locked using a P-controller (ϕ-lock). **b)** Balanced homodyne detection scheme used for detecting the motion along x and y. The light scattered from the particle is combined with a local oscillator and sent to a balanced photo detector. The detected signal is processed by an FPGA and used to apply feedback to the particle's motion. **c)** Schematic of the chip showing the trapping fibers and the planar electrodes. The feedback voltage for the x (y) motion is applied to the horizontal (vertical) electrodes, while the one for the z motion is applied to the central electrode. **d)-e)** Simulated E_x component of the electric field in the xy and xz planes. **f)-g)** Simulated E_y component of the electric field in the xy and yz planes. **h)-i)** Simulated E_z component of the electric field in the xy and xz planes. In **d)-i)** ± 1 V is applied to the respective electrodes. The black lines correspond to the fibers.

where $\vec{E}_1(r,\theta,\phi)$ is the electric field scattered from $\vec{E}_{+x}$ and $\vec{E}_2(r,\theta,\phi)$ the one scattered from $\vec{E}_{-x}$.

Since we are interested in displacement along y we can set x and z to 0. Furthermore, we consider that the foci of

the beams are positioned symmetrically with respect to the origin, i.e., $x_{0,-} = -x_{0,+} = x_0$. We then get:

$$\vec{E}_{sc}(\vec{r}) = e^{iAk_x x_0}\left(\vec{E}_1(r,\theta,\phi) + \vec{E}_2(r,\theta,\phi)\right)e^{-ik_x y_p \sin\theta\sin\phi} = e^{iAk_x x_0}\vec{E}_t(r,\theta,\phi)e^{-ik_x y_p \sin\theta\sin\phi} \tag{5}$$

where $\vec{E}_t(r,\theta,\phi)$ is the electric field scattered from the superposition of both beams and $^{iAk_x x_0}$ is a constant phase factor. At this point, the problem becomes very similar to the one treated in [1], the difference being that instead of the field scattered by a particle we have $\vec{E}_t(r,\theta,\phi)$. Ultimately, the information pattern $\mathcal{I}_y(\theta,\phi)$ is proportional to $|E_t(r,\theta,\phi)|^2\sin^2\theta\sin^2\phi$.

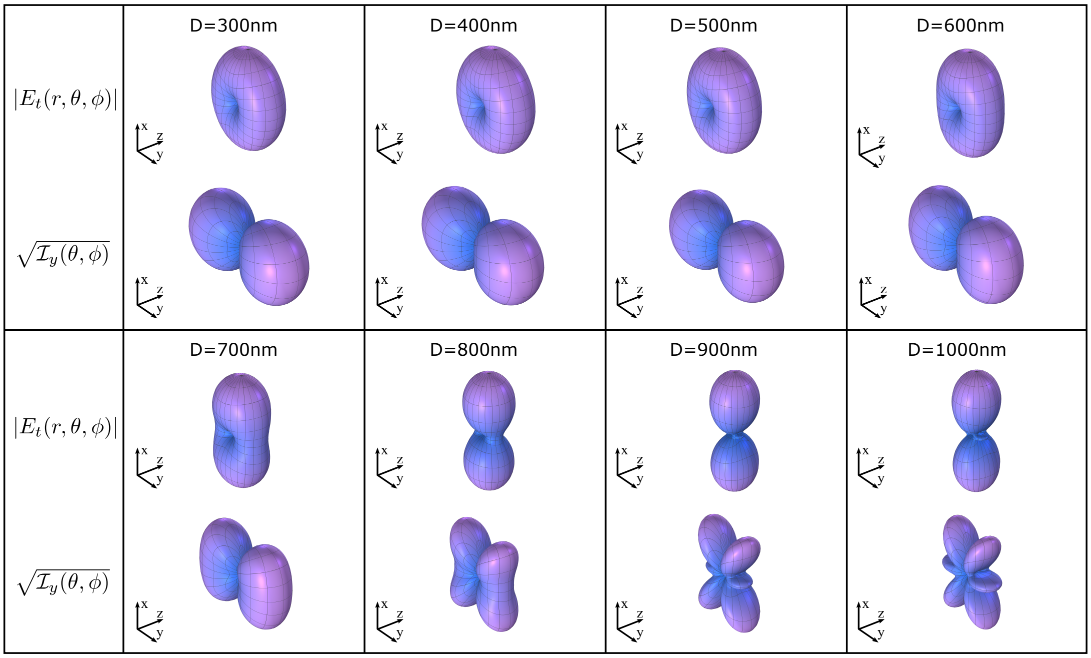

Figure S3. **Scattering and information patterns.** Absolute value of the scattered electric field in the far field by a dielectric particle in a standing wave along x with polarization along z and the corresponding information pattern for the position along y. We look at particles with different diameters to observe the transition from the mode expected from a dipole to a mode that consitst of mulipole contributions. **a** $D = 300$ nm. **b** $D = 400$ nm. **c** $D = 500$ nm. **d** $D = 600$ nm. **e** $D = 700$ nm. **f** $D = 800$ nm. **g** $D = 900$ nm. **h** $D = 1000$ nm.

In Fig. III we show $|E_t(r,\theta,\phi)|$ and $\sqrt{\mathcal{I}_y(\theta,\phi)}$ for silica particles of different diameters for the motion along y. The field was simulated for $\lambda_x = 1064$ nm, $x_0 = 40$ μm and NA = 0.11 ($w_{0,x} = 3.1$ μm). As we can see for diameters up to 700 nm, most information scatters along y. Since we work with a radius of $r = 160$ nm, our use of the fibers along y to detect the motion along y is justified.

IV. COLD DAMPING AND TEMPERATURE MEASUREMENTS

Our experiment follows the approach used in [2]. The ideal linear feedback signal is $F_x^{\mathrm{fb}}(t) \propto \Gamma_x^{\mathrm{fb}}\dot{x}(t) \propto k_x^d \dot{x}(t)$. Due to measurement noise, we can only measure $x'(t) = x(t) + \xi(t)$, where $\xi(t)$ is the detector noise. The real feedback signal is then given by $F_x^{\mathrm{fb}}(t) \propto \Gamma_x^{\mathrm{fb}}\dot{x}'(t) = \Gamma_x^{\mathrm{fb}}(\dot{x}(t) + \dot{\xi}(t))$. The equation of motion can be written as

$$\ddot{x} + \Gamma_m\dot{x} + \Omega_x^2 x = \frac{\sigma\eta(t) + F_x^{\mathrm{fb}}}{m} = \frac{\sigma}{m}\eta(t) - \Gamma_x^{\mathrm{fb}}(\dot{x}(t) + \dot{\xi}(t)) \tag{6}$$

where m is the particle mass, Ω_x is the mechanical eigenfrequency, Γ_m is the mechanical damping term due to the interaction with residual air molecules [3], $\sigma\eta(t)$ is the stochastic force and follows the fluctuation-dissipation theorem [4] where $\sigma = \sqrt{2mk_BT\Gamma_m}$.

The Fourier transform of the time derivative of the measurement noise equals $\mathcal{F}(\dot{\xi}(t)) = \omega\sigma_x$ and $\dot{x}(t)$ is approximated as $\dot{x}(t) \approx -\Omega_x x(t - \pi/(2\Omega_x))$. The power spectral densities (PSDs) of the real position $S_x(\omega)$ and the measured position $S_x^{\mathrm{IL}}(\omega)$ by the in-loop detector are

$$S_x(\omega) = \frac{\sigma^2/m^2}{(\Omega_x^2-\omega^2)^2+(\Gamma_m+\Gamma_x^{\rm fb})^2\omega^2} + \frac{(\Gamma_x^{\rm fb}\omega)^2}{(\Omega_x^2-\omega^2)^2+(\Gamma_m+\Gamma_x^{\rm fb})^2\omega^2}\sigma_x^2, \tag{7}$$

$$S_x^{\rm IL}(\omega) = \frac{\sigma^2/m^2}{(\Omega_x^2-\omega^2)^2+(\Gamma_m+\Gamma_x^{\rm fb})^2\omega^2} + \frac{(\Omega_x^2-\omega^2)^2+\Gamma_m^2\omega^2}{(\Omega_x^2-\omega^2)^2+(\Gamma_m+\Gamma_x^{\rm fb})^2\omega^2}\sigma_x^2. \tag{8}$$

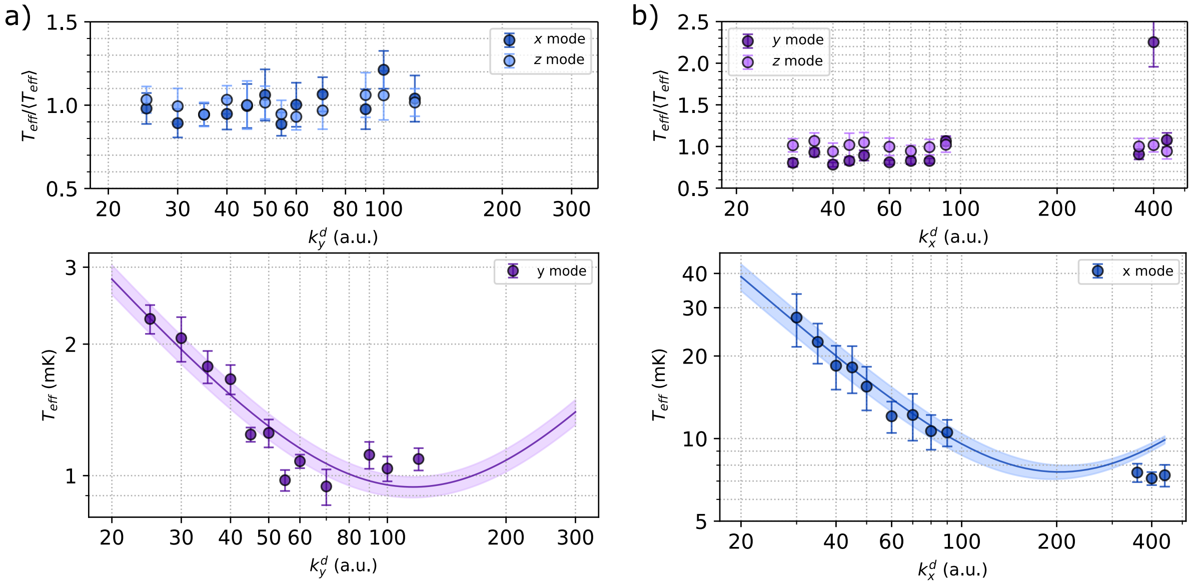

Figure S4. **Effective com temperature of the com versus feedback gain** k_d **a)** Temperature of the com along x (top plot, dark blue points), z (top plot, light blue points) and y (bottom plot, purple points) for increasing values of the feedback gain along y. **b)** Temperature of the com along y (top plot, dark purple points), z (top plot, light purple points) and y (bottom plot, blue points) for increasing values of the feedback gain along x. The plots show that increasing the feedback gain k_d along a certain direction affects mainly the temperature along that same direction. The outlier at kd = 400 is due to spurious dynamic noise from other modes. The temperature is extracted from the measured PSDs based on 10 time traces of 50ms for x,y and 500ms for z, where the cooled mode temperatures are determined from fits to Eq. 8 (bottom) and the uncooled modes by numerical integration across the bandwidth above the noise floor (top). The error bars refer to the standard deviation. Solid lines are fits to Eq. 20 and shaded regions represent the fit uncertainty.

We can relate the particle energy to an effective temperature $T_q^{\rm eff}$ by means of the equipartition and Parseval's theorem [5]. The equipartition theorem describes the mean potential energy of the particle as

$$E_{\rm pot} = \frac{1}{2}m\Omega_m^2\langle x^2\rangle = \frac{1}{2}k_B T_{\rm eff}, \tag{9}$$

where $\langle x^2\rangle$ is the variance of the particle motion along the x direction. Parseval's theorem relates the variance of the position to the integral of the PSD as follows:

$$\langle x^2\rangle = \frac{1}{2\pi}\int_{-\infty}^{\infty} S_x(\omega)d\omega. \tag{10}$$

Combining Eq. 9 and Eq. 10 we can define the effective center-of-mass temperature as

$$T_{\rm eff} = \frac{m\Omega_m^2}{k_B}\frac{1}{2\pi}\int_{-\infty}^{\infty} S_x(\omega)d\omega. \tag{11}$$

Plugging Eq. 8 into Eq. 11 we find

$$T_{\rm eff} = \frac{m\Omega_x^2}{2k_B}\left(\frac{\sigma^2/m^2}{\Omega_x^2(\Gamma_m+\Gamma_x^{\rm fb})} + \frac{(\Gamma_x^{\rm fb}\sigma_x)^2}{\Gamma_m+\Gamma_x^{\rm fb}}\right), \tag{12}$$

The phonon occupation can be approximated as

$$\langle n_x \rangle \approx \frac{k_B T_{\mathrm{eff}}}{\hbar \Omega_x}. \tag{13}$$

The minimum achievable temperature at a pressure p can be calculated using Eq. 12 by solving $\partial T_{\mathrm{eff}}/\partial \Gamma_x^{\mathrm{fb}} = 0$ to find the optimal Γ_x^{fb} and plugging it back in T_{eff}. This gives:

$$T_{\mathrm{eff,min}} = -\frac{m\Gamma_m \sigma_x^2 \Omega_x^2}{k_B} + \frac{\sigma_x \Omega_x}{k_B}\sqrt{\sigma^2 + m^2 \Gamma_m^2 \sigma_x^2 \Omega_x^2} \tag{14}$$

where Γ_m is the mechanical damping at the pressure p.

This minimum temperature can be related to the SNR at a higher pressure p_0 when no feedback is present, given by:

$$\mathrm{SNR}|_{p_0} = \frac{2k_B T}{m\Gamma_{m,0}\Omega_x^2 \sigma_x^2} \tag{15}$$

where $\Gamma_{m,0}$ is the mechanical damping at the pressure p_0. Substituting the expression for the $\mathrm{SNR}|_{p_0}$ into Eq. 14 and using that at low pressures $\Gamma_m \propto p$:

$$T_{\mathrm{eff,min}} = \frac{2T}{\mathrm{SNR}|_{p_0}}\left(-\frac{p}{p_0} + \sqrt{\frac{p}{p_0}\mathrm{SNR}|_{p_0} + \frac{p^2}{p_0^2}}\right) \approx 2T\sqrt{\frac{p}{p_0}\frac{1}{\mathrm{SNR}|_{p_0}}} \tag{16}$$

where the approximation holds for SNR$\gg 1$ and $p/p_0 \ll 1$.

At the bottom of Fig. S4, we show the extracted temperatures for x and y modes at a pressure $p = 5.9 \times 10^{-6}$ mbar and $p = 6.8 \times 10^{-6}$ mbar, respectively. As previously stated, we achieve minimum phonon occupations of $n_x = 1325 \pm 72$ and $n_y = 329 \pm 30$. We measure $\mathrm{SNR}_x = 7.6 \times 10^3$ and $\mathrm{SNR}_y = 4.3 \times 10^5$, both at $p = 14.1$mbar. According to Eq. 16 and using Eq. 13, the minimum achievable phonon occupation are approximately $n_{x,\mathrm{th}} = 830$ and $n_{y,\mathrm{th}} = 220$ phonons in reasonable agreement with the experiment.

We assess the cross-coupling of the feedback signals resulting from inhomogeneous electric fields by measuring the com temperature of the other eigenmodes. As shown at the top of Fig. S4, we find the temperature of the perpendicular eigenmodes unaffected by the gain sweep. This confirms that the cross-coupling is negligible.

In Fig.S5 we show that we are detection-limited by increasing the feedback gain until reaching the characteristic noise-squashing. This behavior is observed for both the x and y axis. We attribute the frequency shift to a not-optimally adjusted delay in the feedback signal[2].

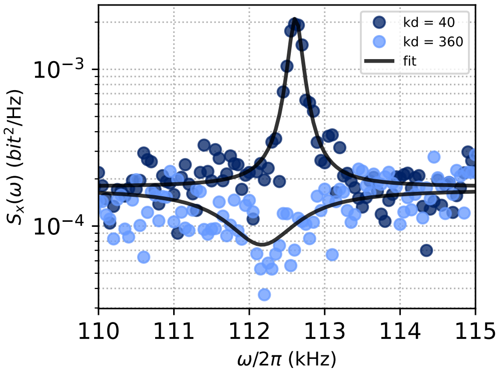

Figure S5. **Noise squashing.** PSDs of the motion along x for two different feedback gains. For $k_x^d = 40$, the motional peak is above the detection noise (dark blue circles), while for $k_x^d = 360$ we observe noise squashing (light blue points). The solid lines are fits to Eq. 8, see SM V for details of the fitting.

V. CALIBRATION AND DATA ANALYSIS

1. *Calibration of the particle displacement*

Here, we review the calibration of the particle displacement $x = \nu_x / c_{cal}$, where ν_x is the detected signal and c_{cal} the calibration factor. We assume the particle is described as a harmonic oscillator at $p = 14$ mbar. The assumption allows us to apply the equipartition theorem and extract c_{cal}.

To calculate the linear calibration factor c_{cal}, we link the expectation value of the potential energy E_{pot} to the variance of the particle displacement $\langle x^2 \rangle$ as

$$E_{pot} = \frac{1}{2} m \Omega_x^2 \langle x^2 \rangle = \frac{1}{2} m \Omega_x^2 \frac{\langle \nu_x^2 \rangle}{c_{cal}^2} = \frac{1}{2} k_B T. \tag{17}$$

where $\langle \nu_x^2 \rangle$ is the variance of the detected signal. Therefore, we can calculate the calibration factor c_{cal} as

$$c_{cal} = \sqrt{\frac{m \Omega_x^2 \langle \nu_x^2 \rangle}{k_B T}}. \tag{18}$$

2. *Extracting the center-of-mass effective temperature*

To extract the temperature of the com motional modes for the x and y directions we fit the in-loop detected signal to the single sided PSD (see Eq. 2 in the main text):

$$S_x^{\mathrm{IL}}(\omega) = \frac{B \Omega_x^2 \Gamma_{\mathrm{eff}}}{(\Omega_x^2 - \omega^2)^2 + \Gamma_{\mathrm{eff}}^2 \omega^2} + \frac{(\Omega_x^2 - \omega^2)^2 + \Gamma_m^2 \omega^2}{(\Omega_x^2 - \omega^2)^2 + \Gamma_{\mathrm{eff}}^2 \omega^2} \sigma_x^2. \tag{19}$$

where the fitting parameters are $\Gamma_{\mathrm{eff}} = \Gamma_m + \Gamma_x^{\mathrm{fb}}$, Γ_m, Ω_x, σ_x and $B = \frac{\sigma^2/m^2}{\Omega_x^2 \Gamma_{\mathrm{eff}}}$.

Then, with the extracted values from the fit, we extract T_{eff} as

$$T_{\mathrm{eff}} = \frac{m \Omega_x^2}{2 k_B} \left(B + \frac{(\Gamma_{\mathrm{eff}} - \Gamma_m)^2 \sigma_x^2}{\Gamma_{\mathrm{eff}}} \right), \tag{20}$$

and convert it to phonon occupations using $\langle n_q \rangle = k_B T_q^{\mathrm{eff}} / \hbar \Omega_q$.

For each value of k_d shown in Fig. 4, we extract the temperature from 10 time traces of 50 ms. The effective temperature, T_{eff}, is calculated as the average of the 10 temperatures extracted, and the error bars are given by the standard deviation.

The solid line in Fig. 4, is a fit to Eq. 12, where m is extracted from the calibration time trace at 14 mbar, Γ_m, σ, σ_x and Ω_x are taken as the average of the values obtained by fitting each of the time traces of 50 ms and the proportionality factor between k_d and Γ_x^{fb} is the only remaining parameter. The shaded region corresponds to Eq. 12 when plugging in the uncertainties in Γ_m, σ, σ_x and Ω_x, which are calculated as the standard deviation in the values extracted from each of the time traces of 50 ms.

VI. SENSING WITH A COLD DAMPED HARMONIC OSCILLATOR

Consider the following equation for a driven harmonic oscillator under linear feedback cooling:

$$\ddot{x} + (\Gamma_m + \Gamma_x^{\mathrm{fb}}) \dot{x} + \Omega_x^2 x = \frac{\sigma}{m} \eta(t) + \Gamma_x^{\mathrm{fb}} \dot{\xi}(t) + \frac{F(t)}{m} \tag{21}$$

where $\Gamma_m + \Gamma_x^{\mathrm{fb}} = \Gamma_{\mathrm{eff}}$ is the effective damping and $F(t)$ is an external force that we want to measure.

The response of the system to external driving is given by the transfer function of the harmonic oscillator $H(\omega)$ defined as

$$H(\omega) = \frac{1/m}{\Omega_x^2 - \omega^2 + i\omega(\Gamma_m + \Gamma_x^{\mathrm{fb}})}. \tag{22}$$

The real displacement PSD of the driven harmonic oscillator under linear feedback cooling is given as

$$S_x(\omega) = |H(\omega)|^2\sigma^2 + |H(\omega)|^2(m\Gamma_x^{\mathrm{fb}}\sigma_x\omega)^2 + |H(\omega)|^2 S_f, \tag{23}$$

where σ^2, σ_x^2 and S_f are the PSDs related to the thermal, measurement and external force noise [6], respectively, all of which we consider Gaussian white noise.

To estimate the SNR for real force measurements we need to consider the in-loop PSD from Eq. 8

$$S_x^{\mathrm{IL}}(\omega) = |H(\omega)|^2\sigma^2 + |H(\omega)|^2 m^2\sigma_x^2[(\Omega_x^2 - \omega^2)^2 + \Gamma_m^2\omega^2] + |H(\omega)|^2 S_f. \tag{24}$$

From Eq. 24 we estimate

$$|H(\omega)|^{-2}S_x^{\mathrm{IL}}(\omega) = S_f + \sigma^2 + m^2\sigma_x^2[(\Omega_x^2 - \omega^2)^2 + \Gamma_m^2\omega^2], \tag{25}$$

To get a minimum measurable force, we need to overcome noise present in the system. So we are interested in a minimum signal-to-noise (SNR) greater than one, which is defined as

$$\mathrm{SNR} = \sqrt{\frac{S_f}{\sigma^2 + m^2\sigma_x^2[(\Omega_x^2 - \omega^2)^2 + \Gamma_m^2\omega^2]}} > 1 \tag{26}$$

We then obtain that the force sensitivity as

$$\sqrt{S_f(\omega)} = \sqrt{\sigma^2 + m^2\sigma_x^2[(\Omega_x^2 - \omega^2)^2 + \Gamma_m^2\omega^2]} \tag{27}$$

We obtain $\sqrt{S_f(\omega)}$ by measuring $S_x^{\mathrm{IL}}(\omega)$ and multiplying it by $|H(\omega)|^{-2}$ using the parameters extracted from the fit of the IL PSD. Evaluating Eq. 26 at resonance ($\omega = \Omega_x$) yields the minimum achievable force sensitivity

$$\sqrt{S_f(\Omega_x)} > \sqrt{2mk_BT\Gamma_m + m^2\Omega_x^2\Gamma_m^2\sigma_x^2} \tag{28}$$

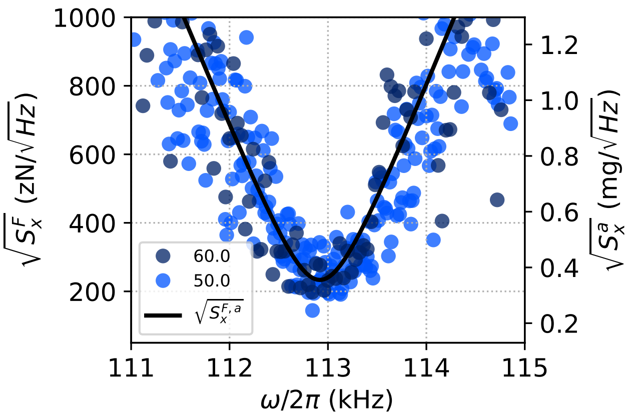

Figure S6. **Force and acceleration sensitivity. a)** Force spectral sensitivity and **b)** Acceleration spectral sensitivity of the motion along x for two different feedback gains. The solid black line corresponds to Eq. 27 using the parameters extracted from the fitted IL PSDs.

In Fig. S6 we show the force and acceleration PSDs for the x and y modes, confirming the sensitivity is independent of the feedback gain and agrees with the expected sensitivities from Eq. 28. Note that, the sensitivity is always deteriorated by the detection noise (see Eq. 26).

Let us now consider an out-of-loop detector (ool) for sensing purposes [2]. The PSD for a driven harmonic oscillator with cold damping will be

$$S_x^{\text{ool}}(\omega) = \frac{\sigma^2/m^2}{(\Omega_x^2-\omega^2)^2+(\Gamma_m+\Gamma_x^{\text{fb}})^2\omega^2} + \frac{(\Gamma_x^{\text{fb}}\omega)^2}{(\Omega_x^2-\omega^2)^2+(\Gamma_m+\Gamma_x^{\text{fb}})^2\omega^2}\sigma_x^2 + \frac{S_f/m^2}{(\Omega_x^2-\omega^2)^2+(\Gamma_m+\Gamma_x^{\text{fb}})^2\omega^2} + \sigma_\beta^2, \quad (29)$$

such that

$$|H(\omega)|^{-2} S_x^{\text{ool}}(\omega) = \sigma^2 + m^2(\Gamma_x^{\text{fb}}\omega)^2\sigma_x^2 + S_f + |H(\omega)|^{-2}\sigma_\beta^2 \quad (30)$$

where σ_β is related to the variance of the out-of-loop detector noise. If we are interested in having a SNR>1, we obtain an out-of-loop sensitivity on resonance:

$$\sqrt{S_f^{\text{ool}}} > \sqrt{2mk_BT\Gamma_m + m^2\Omega_x^2[(\Gamma_x^{\text{fb}}\sigma_x)^2 + (\Gamma_m+\Gamma_x^{\text{fb}})^2\sigma_\beta^2]}, \quad (31)$$

where $\sqrt{S_f^{\text{ool}}}$ is the out-of-loop force sensitivity. Eq. 31 includes an additional dependency on the out-of-loop detection noise σ_β^2. If we are well above the detector noise floor, σ_x^2 and σ_β^2 are very small and the thermal noise limited assumption is justified. However, if our signal is comparable to the noise floor, Eq. 31 has to be considered. In the latter case, the out-of-loop detection will also have higher noise contributions than the in-loop detection.

VII. SURFACE NOISE HEATING

Surface noise heating due to electric field noise acting on charged particles is a well known decoherence source in ion traps [7]. In the following section we estimate the expected noise levels on the hybrid chip due to exposed gold electrodes and dielectric fiber facets. We base our estimation on [8, 9].
We consider a nanoparticle of radius $R = 160$nm with $n_q = 100$ elementary charges to be levitating above a planar gold electrode covered by a thin dielectric layer of thickness t_d, composed of oxides or hydrocarbon compounds. The distance between the electrode and nanoparticle equals $d_z = 203\mu$m. The distance to the dielectric fiber facet yields $d_x/2 = 40\mu$m.

The PSD of the eletric-field fluctuations $S_E^\perp$ translates to a fluctuating force S_F as $S_E^\perp = \frac{S_F}{q^2}$. The fluctuation–dissipation theorem then again links S_F to a dissipative damping $\Gamma(\omega)$, such that $S_F(\omega) = 2k_BTm\Gamma(\omega)$.

The PSD of the electric field noise perpendicular to an infinite surface is given by [8]

$$S_E^\perp = \frac{3}{8\pi}\frac{\tan\theta}{\epsilon(1+\tan^2\theta)}\frac{k_BTt_d}{\omega d^4} \quad (32)$$

The thin dielectric layer at temperature T is characterized by complex permittivity $\epsilon' = \epsilon(1+j\tan\theta)$ with the loss tangent assumed to be small $\tan\theta < 1$. We assume the upper limit for the dielectric thickness formed by hydrocarbon compounds $t_d \approx 2$nm with $\epsilon = 2\epsilon_0$ and $\tan\theta \sim 0.01$ [8].

Fig. S7a displays S_F due to the gold electrodes (solid blue line) depending on the distance d_z. For comparison we plot the expected thermal force noise stemming from residual gas collisions at pressure $p = 9\times10^{-6}$mbar (dashed purple line). At $d_z = 203\mu$m (solid purple line) we estimate $S_F = 3\times10^{-46}\text{N}^2/\text{Hz}$, which is negligible in comparison to the thermal force noise at $S_{F\,th} = 2k_BTm\Gamma_m = 6\times10^{-39}\text{N}^2/\text{Hz}$. The electric field noise becomes relevant for distances below $d_z < 3\mu$m or $p < 10^{-12}$mbar.

The electric field noise stemming from the dielectric fiber facets we treat slightly different. For simplicity we still assume the dielectric surface to be infinitely large, which is a coarse assumption given a fiber diameter of 120μm and a nanoparticle radius $R = 160$nm at a distance $d_x = 40\mu$m. We assume for SiO_2 the following values $\epsilon = 3.9\epsilon_0$ and $\tan\theta \sim 0.0013$ [9]. We divide the fiber into slabs of thickness $t_d = 1\mu$m at varying d_i and calculate their individual contribution to $S_E^\perp$. The individual contributions are then summed up as

$$\Sigma S_{F\perp} = \Sigma_{i=1}^N S_E^\perp(d_i)q^2 \quad (33)$$

with $\delta d = \Sigma_{i=1}^N d_i$. Fig. S7b displays ΣS_F for varying dielectric thickness. We observe a saturation of the force noise level to $\Sigma S_{F\perp} = 3\times10^{-41}\text{N}^2/\text{Hz}$ beyond $\delta d = 100\mu$m, where additional dielectric slabs have very little impact on the overall noise level. This corresponds to a noise level well below the thermal contribution $S_{F\,th}$, but would be come relevant at pressures $p \approx 10^{-7}$mbar. For more precise estimations, one would need to take the 3D fibre shape into account.

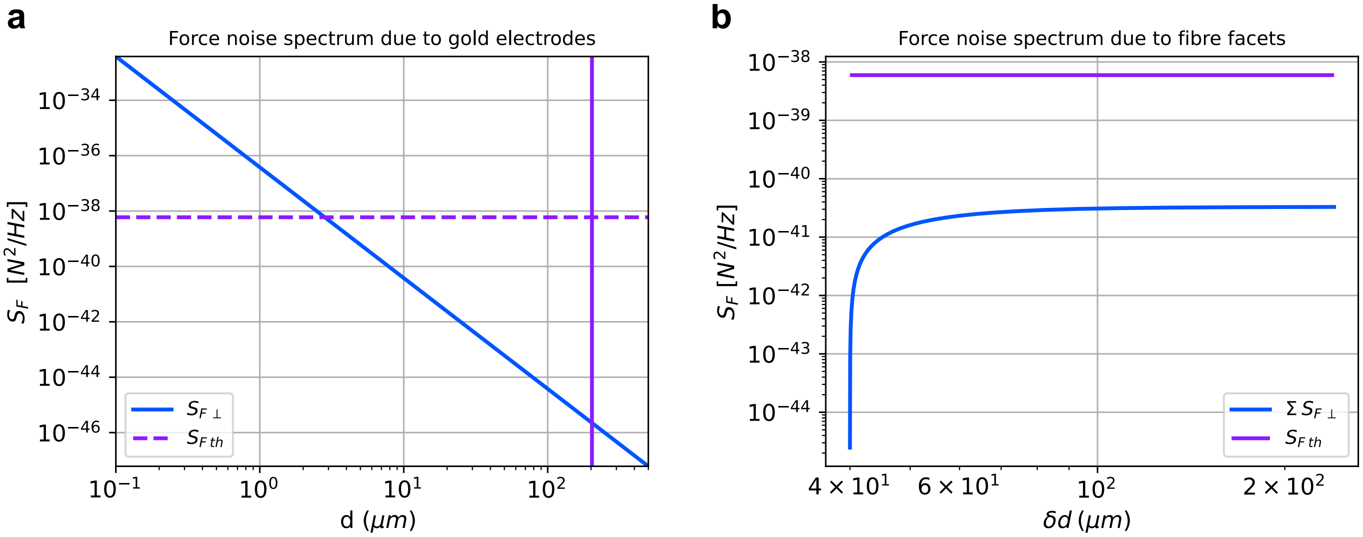

Figure S7. **Surface noise force** in comparison to the thermal force noise due to **a)** gold electrodes and **b)** a dielectric fiber facet

[1] F. Tebbenjohanns, M. Frimmer, and L. Novotny, Physical Review A **100**, 043821 (2019).
[2] G. P. Conangla, F. Ricci, M. T. Cuairan, A. W. Schell, N. Meyer, and R. Quidant, Phys. Rev. Lett. **122**, 223602 (2019).
[3] T. Li, S. Kheifets, and M. G. Raizen, Nature Phys **7**, 10.1038/nphys1952 (2011).
[4] R. Kubo, Reports on progress in physics **29**, 255 (1966).
[5] M.-A. Parseval, Mém. prés. par divers savants, Acad. des Sciences, Paris,(1) **1**, 638 (1806).
[6] F. Ricci, M. T. Cuairan, G. P. Conangla, A. W. Schell, and R. Quidant, Nano Lett. **19**, 6711 (2019).
[7] M. Brownnutt, M. Kumph, P. Rabl, and R. Blatt, Reviews of modern Physics **87**, 1419 (2015).
[8] M. Kumph, C. Henkel, P. Rabl, M. Brownnutt, and R. Blatt, New Journal of Physics **18**, 023020 (2016).
[9] M. Teller, D. A. Fioretto, P. C. Holz, P. Schindler, V. Messerer, K. Schüppert, Y. Zou, R. Blatt, J. Chiaverini, J. Sage, *et al.*, Physical Review Letters **126**, 230505 (2021).
